# Lecithine in Tuberculosis Apropos of a Communication by M. Bouchard to the Academy of Science

**Published:** 1903-11

**Authors:** 

**Affiliations:** Revue Crititique de Medicine et de Chiruir


### Lecithine in Tuberculosis Apropos of a Communication by M. Bouchard to the Academy of Science.

Professor Bouchard has just presented to the Academy of Sciences a note resuming the researches of MM. Claude and Zaki on the action of Lecithine in tuberculosis.

These gentlemen have tried to determine more accurately than by clinical experimentation the action of Lecithine on tuberculized organs. These researches have been made on animals and on man.

I. *Experimental Researches*.—They include two series of experiments:

1. Three guinea pigs were inoculated with tuberculosis products. Two received injections of Lecithine oil (0 gr. 10 of Lecithine daily); the third used as witness. The last one died in thirty-five days. Of the two former, one died three and the other five weeks later. The analysis of the urines showed that under the influence of Lecithine, the tuberculized animals eliminated less phosphorus (0.13 instead of 0.32 in the average) and that their nutrition was better, as the coefficient $\frac{Az\,U}{Az\,T}$ coefficient of utilization of azote, remained on them in the neighborhood of 0.90 while it fell to 0.78 on the witness.

2. Three lots of three guinea pigs each were similarly inoculated with the bacillus of Koch. One lot received subcutaneous injections of Lecithine oil (0 gr. 05 of Lecithine daily), another lot absorbed in the stomach 0 gr. 05 of Lecithine. The third lot took no Lecithine and was used as witness.

All the animals of the last lot died within fifty days; of the two other lots who were well tuberculized, two animals of each lot are yet living on September first—that is, nearly three months after tuberculosis inoculation—and their weight has only begun to decrease. Nutrition among them is better than it was among the witnesses, the coefficient $\frac{Az\ U}{Az\ T}$ is superior to the one observed on the witnesses and the elimination of phosphates, recorded among the last one is 0.45 on the average, while it is only 0.27 and 0.02 among the animals of the two lots which have received Lecithine.

II. *Researches on Man.*—They were made at the hospital Saint Antoine. The authors have recorded twenty observations concerning cases of pulmonary tuberculosis at various degrees.

On the eight tuberculous patients, at the beginning, or in the first stage of the disease, the results of the treatment with Lecithine were most satisfactory. The general condition, bad or only languid, changed in a few days, strength increased, appetite reappeared almost immediately and the weight increased in considerable proportions, 2, 3 and even 7 kilogrammes in less than twenty days. The decrease of the amount of phosphates in the urine followed almost immediately after ingestion of Lecithine; 1.88 to 1.08 average in one case; 1.40 to 0.99 in another; 2.55 to 2.03 in a third one, etc. Finally the coefficient of utilization of azote $\frac{Az\ U}{Az\ T}$ increases constantly and in various proportions, according to the cases, from 0.70 to 0.90 average in observation VI; from 0.77 to 0.82 in observation V; from 0.79 to 0.85 in observation III, etc.

Consequently under the influence of the stimulation of the appetite provoked by Lecithine not only the patients absorb and eliminate more, as it is demonstrated by the amount of urea disclosed by the analysis, but they elaborate better and the organic combustions are remarkably enhanced.

Five observations of pulmonary tuberculosis in the second stage show also that Lecithine gives very satisfactory results. In all these cases we have noticed during the ingestion of Lecithine an increase of weight among the subjects who were previously getting lean. Sometimes the increase in weight was stopped by hectic fever or intercurrent complications but it was only temporary. Besides, in all these patients the analysis of the urine showed us a diminution of the elimination of phosphates and an increase of the coefficient $\frac{Az\ U}{Az\ T}$.

In four tuberculous patients with large cavities, the results varied. An apyretic patient received from Lecithine the same benefit derived by the others as described before.

Three other patients affected with fever, with great oscillations, and having various general troubles, kept losing weight. But, and this is most important, the elimination of phosphates decreased since the use of Lecithine, while the coefficient $\frac{Az\ U}{Az\ T}$ increased slightly. There was no modification in the evolution of the lesions. We must state, however, that our observations covered only a short space of time and we were not able on that account to appreciate a change in the alternations of the organic lesions so far advanced.

The same remarks could be applied to two cases of acute pulmonary phthisis (broncho-pneumonic form). The loss of weight did not stop after the administration of Lecithine, but it was less rapid than before, the general condition at times greatly improved, the elimination of phosphates, as well as the coefficient $\frac{Az\ U}{Az\ T}$ were favorably influenced as in the previous observations, notwithstanding the poor condition of the organism.

On a young man who was apparently at the beginning of an attack of acute phthisis, the physical signs and the general phenomena were suddenly modified as soon as Lecithine was administered, notwithstanding a tendency toward a relapse, when the dose of Lecithine was increased from 30 centigrammes to 50 centigrammes; the young man left the hospital in a fairly good condition, having only a few traces of lesions at one of the summits of the lungs.

Finally, in our last observation concerning a case of granulitis treated with Lecithine at a dose of 50 centigrammes two days before death, we noticed, notwithstanding the ultimate cachetic state of the patient, a diminution of the elimination of the phosphates after the ingestion of the medicine.

From these researches we may conclude that Lecithine, on account of its specific action on the elimination of the phosphates through the urines, and of its influence on the nutritive exchanges (increase of the coefficient $\frac{Az\ U}{Az\ T}$ or utilization of azote) may be considered as a precious adjunct to the various methods of treatment of tuberculosis.

This communication appeared to us to be very important because it demonstrates the efficacy of the therapeutical action of Lecithine in the treatment of tuberculosis. We have at the present time under treatment eight tuberculous patients to whom we give Lecithine.

We have administered the medicine under different forms. In three cases where the gastric tolerance exists in its normal condition, we give five Clin pills daily. For a twelve-year-old child affected with tuberculous cervical adenopathy we have advised granulated Lecithine. In the other four cases characterized by an advanced stadium of the bacillary evolution we give hypodermic injections with Clin solution. The injections are made every other day according to the modus operandi previously described. The dose of one cubic centimeter of the oily solution containg five centigrammes of Lecithine is generally well tolerated. We may publish later the results of these eight observations.

DR. RAYMOND,
(Revue Crititique de Medicine et de Chiruir.)

